# The MSIS-29 and SF-36 as outcomes in secondary progressive MS trials

**DOI:** 10.1177/13524585221105465

**Published:** 2022-07-25

**Authors:** Eva MM Strijbis, Pavle Repovic, Jop Mostert, James D Bowen, Bernard MJ Uitdehaag, Gary Cutter, Marcus W Koch

**Affiliations:** Department of Neurology, MS Center Amsterdam, Amsterdam University Medical Centers, Amsterdam, The Netherlands; Multiple Sclerosis Center, Swedish Neuroscience Institute, Seattle, WA, USA; Department of Neurology, Rijnstate Hospital, Arnhem, The Netherlands; Multiple Sclerosis Center, Swedish Neuroscience Institute, Seattle, WA, USA; Department of Neurology, MS Center Amsterdam, Amsterdam University Medical Centers, Amsterdam, The Netherlands; Department of Biostatistics, The University of Alabama at Birmingham, Birmingham, AL, USA; Departments of Clinical Neurosciences and Community Health Sciences, University of Calgary, Calgary, AB, Canada

**Keywords:** Secondary progressive MS, patient-reported outcome measures, health related quality of life (HRQOL), MSIS-29, SF-36

## Abstract

**Background:**

Patient-reported outcome measures (PROMs) are often used in clinical research, but little is known about their performance as longitudinal outcomes.

**Methods:**

We used data from ASCEND, a large SPMS trial (*n* = 889), to investigate changes on the Short Form Health Survey 36 (SF-36 v2) and the Multiple Sclerosis Impact Scale (MSIS-29) over 2 years of follow-up.

**Results:**

PROM scores changed little over the 2 years of follow-up. In contrast to physical disability measures, there was no consistent trend in PROM change: significant worsening occurred about as often as improvement. Using a 6-month confirmation reduced the number of both worsening and improvement events without altering their relative balance. There was no clear difference in worsening events in groups based on population characteristics, nor was there a noticeable effect using different thresholds for clinically significant change.

**Conclusion:**

We found little consistent change in MSIS-29 and SF-36 over 2 years of follow-up in people with SPMS. Our findings show a disconnect between disability worsening and PROM change in this population. Our findings raise caution about the use of these PROMs as primary outcome measures in SPMS trials and call for a critical reappraisal of the longitudinal use of these measures in SPMS trials.

## Introduction

Patient-reported outcome measures (PROMs) are frequently used to evaluate patients’ perspectives on disability, wellbeing, and the impact of disease. These constructs fall under the overarching concept of health-related quality of life (HRQOL). The two most commonly used measures of HRQOL in multiple sclerosis (MS) are the Medical Outcomes Study Short Form Health Survey (SF-36)^1^ and the Multiple Sclerosis Impact Scale (MSIS-29).^2^ The SF-36 investigates physical and social functioning using eight subscales and has been used in many diseases. The SF-36 can be summarised into two summary scores, the mental health component score (MCS) and the physical health component score (PCS).^3^ The MSIS-29 was developed as an MS-specific PROM. The MSIS-29 is a 29-item questionnaire measuring the perceived impact of disability on activities of daily living and wellbeing. The MSIS-29 similarly can be summarised in two scores: the physical (MSIS-Physical) and psychological (MSIS-Psychological) summary score.

Both of these PROMs are often used as secondary outcomes in clinical trials, in value-based health care initiatives, and even in marketing authorisation procedures for investigated compounds in all forms of MS. Both instruments have good psychometric properties in a cross-sectional context, but their usefulness as longitudinal outcome measures in MS is not well established, especially across the spectrum of MS subtypes.

In this investigation, we used data from a large phase III randomised controlled trial in secondary-progressive multiple sclerosis (SPMS) to describe longitudinal change on these measures and to determine their usefulness as outcomes in the setting of a clinical trial. We investigated significant worsening and similarly defined improvement in these PROMs over 2 years of follow-up and compared unconfirmed and confirmed significant change. We also investigated how baseline factors such as sex, treatment arm, or disability at baseline impact worsening, and explored different threshold definitions for clinically significant change.

## Methods

### ASCEND data set

ASCEND was a randomised, double-blind, placebo-controlled, two-arm trial of natalizumab treatment in SPMS.^4^ The inclusion criteria were age 18 to 58 years inclusive, SPMS for 2 or more years, disability progression over the previous year, a screening Expanded Disability Status Scale (EDSS)^5^ score of 3.0 to 6.5 inclusive, and a Multiple Sclerosis Severity Score (MSSS)^6^ of 4 or more. Patients with a clinical relapse in the 3 months before inclusion were excluded, as were patients with a timed-25-foot walk test (T25FW)^7^ of more than 30 seconds. In ASCEND, SPMS was defined as relapsing-remitting disease followed by progressive disability independent of or not explained by MS relapses for at least 2 years prior to inclusion. The trial did not show a treatment benefit of natalizumab over placebo.

### SF-36 and MSIS-29 assessments

In ASCEND, trial participants completed MSIS-29 and SF-36 (v2) questionnaires at baseline, and then at 24, 48, 72, and 96 weeks. For this analysis, we calculated MSIS-29 Physical and Psychological scores for each time point. MSIS-29 Psychological and Physical scores can range from 0 to 100, with higher scores indicating worse HRQOL.^2^ We calculated SF-36 Physical Component Summary (PCS) and Mental Component Summary (MCS) scores for each of these time points. SF-36 PCS and MCS scores range from 0 to 100, with higher scores indicating better HRQOL.^1,3^ For the MSIS-29 Physical and Psychological scores, we defined significant worsening as an increase by 8 or more points compared to baseline.^8,9^ For the SF-36 PCS and MCS scores, we defined a 5-point or more decrease from baseline as significant worsening.^10,11^ To compare PROMs with physical disability worsening, we chose the physical disability outcomes EDSS and the T25FW. We defined significant worsening on the EDSS as an increase by one whole point if the comparator EDSS was 5.5 or lower, and by one-half point if the comparator EDSS was 6.0 or 6.5. We defined significant worsening on the T25FW as a 20% increase in the time needed to complete the T25FW (average of two trials).^12^ We also explored the association of the baseline characteristics sex, EDSS at baseline, and trial arm with worsening on the PROMs during follow-up.

### Statistical analysis

#### Average change in HRQOL over time

We calculated the change in PROM summary scores between baseline and each trial visit.

#### Proportion of patients with unconfirmed and confirmed clinically significant change

We calculated the percentage of patients with unconfirmed, 6 months, and 12 months confirmed significant change (improvement or worsening) in HRQOL at each visit compared to baseline. To explore changes in HRQOL occurring between later time points in the study, we calculated the percentage of patients with significant HRQOL change between baseline and 24 weeks, between 24 and 48 weeks, between 48 and 72 weeks, and between 72 and 96 weeks of follow-up. We used Student’s *t*-tests to compare the change in PROM summary scores between patients with and without significant worsening of the EDSS and T25FW in these same intervals.

#### Effect of different thresholds of the definition of significant change

To explore the importance of the definition of the threshold for significant change, we calculated the proportions of patients with HRQOL change at different cut-off scores. In addition to the generally used threshold of 8 points for the MSIS, we explored ‘any change’, ‘4 point’ and ‘16 point’ thresholds for MSIS Physical and MSIS Psychological scores. In addition to the generally used 5-point threshold for the SF-36, we explored ‘any change’, ‘2 point’ and ‘10 point’ thresholds for the SF-36 PCS and MCS scores.

#### Association of baseline characteristics and significant PROMs worsening

We used contingency tables and chi-square tests to investigate the associations of the baseline characteristics, sex, EDSS at baseline, and treatment arm with significant PROM worsening. We used the R statistical software package version 4.0.5 for all statistical analyses.^13^ Statistical significance was understood to be at the two-tailed 0.05 level.

#### Data availability

The data used in this study is available upon request from Biogen. Individual participant data collected during the trial is shared after anonymization and on approval of a research proposal and data sharing agreement. Research proposals can be submitted online (www.biogenclinicaldatarequest.com).

## Results

### ASCEND data set

The ASCEND data set contained data on 889 patients. Table 1 shows their baseline characteristics.

**Table 1.** Baseline clinical, imaging and HRQOL characteristics at screening in the ASCEND data set. Higher scores on MSIS-29 indicate worse HRQOL, higher scores on the SF-36 indicate better HRQOL.

Descriptives	*N*	ASCEND
Number of participants	889	
Sex (f/m, %)	889	550 (61.9%)/339 (38.1%)
Age (mean, SD)	889	47.2 (7.6)
Disease duration prior at baseline (mean, SD)	889	16.4 (7.7)
MSIS-29 Physical score (mean, SD)	866	50.8.(20.2)
MSIS-29 Psychological score (mean, SD)	876	39.1 (22.4)
SF-36 PCS score (mean, SD)	846	33.3 (7.9)
SF-36 MCS score (mean, SD)	846	47.0 (10.6)
EDSS (median, IQR)	889	6.0 (5.0 to 6.5)
T25FW (median, IQR)	885	11.2 (7.9 to 17.0)
NHPT (median, IQR)	852	30.3 (25.5 to 38.8)
SDMT (median, IQR)	844	39 (30 to 49)

SD: standard deviation; MSIS: Multiple Sclerosis Impact Scale; EDSS: Expanded Disability Status Scale; IQR: interquartile range; PCS: Physical Component Summary; MCS: Mental Component Summary; NHPT: Nine Hole Peg Test; SDMT: Symbol Digit Modalities Test.

### Change in HRQOL summary scores

**Figure 1.**
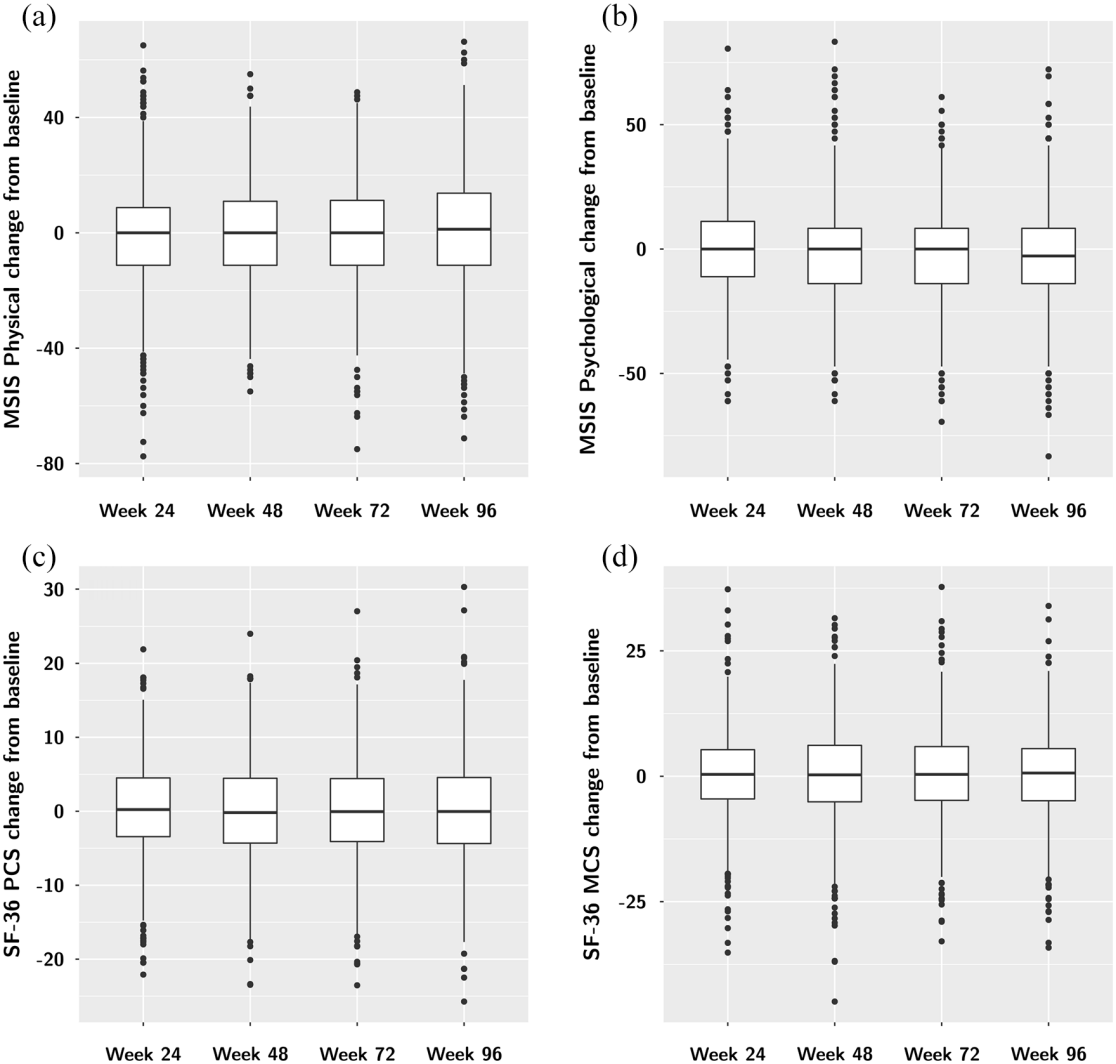
Change from baseline in the four HRQOL summary scores: MSIS Physical (a), MSIS Psychological (b), SF-36 PCS (c), and SF-36 MCS (d). There is overall little change in all four investigated summary scores over the 96 weeks of follow-up in ASCEND. PCS: Physical Component Summary, MCS: Mental Component Summary.

SF-36 and MSIS-29 summary scores changed little over the 2-year duration of this trial. The MSIS-29 physical at baseline was 50.8 (SD 20.2) compared to 50.5 (SD 23.3) at week 96. The MSIS-29 psychological score was 39.1 (SD 22.4) at baseline, compared to 36.7 (SD 23.9) at week 96. The SF-36 PCS was 33.3 (SD 7.9) at baseline, compared to 33.5 (SD 8.6) at week 96, and the SF-36 MCS was 47.0 (SD 10.6) at baseline, compared to 47.7 (10.7) at week 96.^14^ There was little overall change in PROM summary scores from baseline throughout the trial (Figure 1). The median change in all summary scores was about equal in the positive and the negative direction and averaged approximately zero (Figure 1).

### Significant PROM change over time

**Figure 2.**
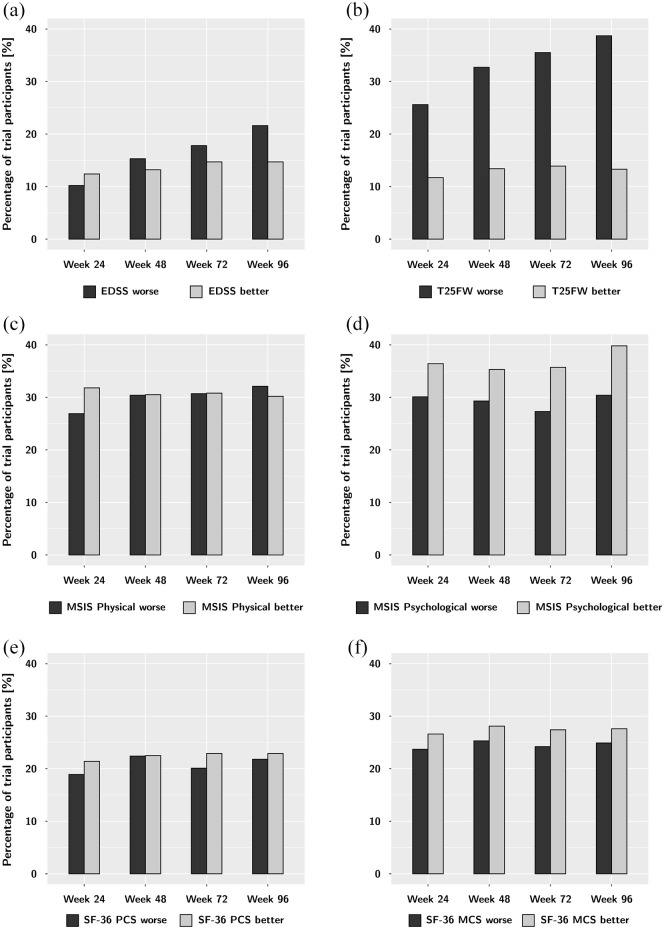
Significant unconfirmed worsening and improvement on the EDSS (a), T25FW (b) and the four HRQOL summary scores: MSIS Physical (c), MSIS Psychological (dSF), -36 PCS (eSF), and -36 MCS (f) over the course of the trial compared to baseline. While the disability outcomes EDSS and T25FW show a steady increase in worsening events throughout follow-up, there is little change in and no consistent trend in the HRQOL measures. Throughout the trial, participants were at least as likely to report improvement as worsening in HRQOL.

For the MSIS-29 physical, the percentage of patients with unconfirmed significant worsening increased slightly but steadily throughout the trial, from 26.9% at week 24% to 32.1% at week 96 (Table 2 and Figure 2). Worsening on the other PROM summary scores did not show a consistent pattern, and was quite stable over the course of follow-up. Remarkably, the proportion of participants with significant improvement on the PROM summary scores compared to baseline was similar or higher compared to those with significant worsening (Table 2). These findings on PROM worsening stand in contrast to worsening on the physical disability measures EDSS and T25FW, which showed a steady increase in worsening events (Table 2 and Figure 2).

**Table 2.** Proportion of patients with significant change (worsening or improvement) in EDSS, T25FW and HRQOL measures over 2 years of follow-up compared to the baseline visit.

Participants with unconfirmed (U) worsening or improvement (%)	24 weeks	48 weeks	72 weeks	96 weeks
EDSS worse U (*n* (%))	82 (10.2)	114 (15.3)	122 (17.8)	140 (21.6)
EDSS better U (*n* (%))	100 (12.4)	98 (13.2)	101 (14.7)	95 (14.7)
T25FW worse U (*n* (%))	202 (25.6)	235 (32.7)	232 (35.5)	236 (38.7)
T25FW better U (*n* (%))	92 (11.7)	96 (13.4)	91 (13.9)	81 (13.3)
MSIS-29 Physical worse U (*n* (%))	210 (26.9)	218 (30.4)	205 (30.7)	203 (32.1)
MSIS-29 Physical better U (*n* (%))	248 (31.8)	219 (30.5)	206 (30.8)	191 (30.2)
MSIS-29 Psychological worse U (*n* (%))	239 (30.1)	216 (29.3)	185 (27.3)	195 (30.4)
MSIS-29 Psychological better U (*n* (%))	289 (36.4)	260 (35.3)	242 (35.7)	255 (39.8)
SF-36 PCS worse U (*n* (%))	139 (18.9)	152 (22.4)	126 (20.1)	130 (21.8)
SF-36 PCS better U (*n* (%))	158 (21.4)	153 (22.5)	144 (22.9)	136 (22.9)
SF-36 MCS worse U (*n* (%))	175 (23.7)	172 (25.3)	152 (24.2)	148 (24.9)
SF-36 MCS better U (*n* (%))	196 (26.6)	191 (28.1)	172 (27.4)	164 (27.6)
Participants with confirmed (C) worsening or improvement (%)	24 weeks	48 weeks	72 weeks	
EDSS worse 6M C (*n* (%))	57 (7.8)	76 (11.2)	91 (14.1)	
EDSS better 6M C (*n* (%))	59 (8.0)	67 (9.9)	75 (11.6)	
T25FW worse 6M C (*n* (%))	134 (19.0)	155 (24.0)	148 (24.6)	
T25FW better 6M C (*n* (%))	49 (7.0)	57 (8.8)	59 (9.8)	
MSIS-29 Physical worse 6M C (*n* (%))	111 (15.8)	119 (18.2)	129 (20.5)	
MSIS-29 Physical better 6M C (*n* (%))	142 (20.2)	133 (20.3)	137 (21.7)	
MSIS-29 Psychological worse 6M C (*n* (%))	122 (16.8)	109 (16.2)	103 (16.1)	
MSIS-29 Psychological better 6M C (*n* (%))	178 (24.5)	172 (25.5)	170 (26.6)	
SF-36 PCS worse 6M C (*n* (%))	74 (11.4)	71 (11.8)	65 (11.4)	
SF-36 PCS better 6M C (*n* (%))	65 (10.0)	69 (11.5)	78 (13.7)	
SF-36 MCS worse 6M C (*n* (%))	80 (12.3)	76 (12.6)	72 (12.6)	
SF-36 MCS better 6M C (*n* (%))	102 (15.7)	108 (18.0)	101 (17.7)	
MSIS-29 Physical worse 12M C (*n* (%))	109 (16.0)	114 (17.8)		
MSIS-29 Physical better 12M C (*n* (%))	123 (17.9)	123 (19.0)		
MSIS-29 Psychological worse 12M C (*n* (%))	98 (13.9)	108 (16.4)		
MSIS-29 Psychological better 12M C (*n* (%))	157 (22.3)	162 (24.6)		
SF-36 PCS worse 12M C (*n* (%))	55 (8.7)	69 (11.8)		
SF-36 PCS better 12M C (*n* (%))	71 (11.1)	70 (11.8)		
SF-36 MCS worse 12M C (*n* (%))	70 (11.1)	59 (10.1)		
SF-36 MCS better 12M C (*n* (%))	89 (14.0)	97 (16.6)		

EDSS: Expanded Disability Status Scale; MSIS: Multiple Sclerosis Impact Scale; PCS: Physical Component Summary; MCS: Mental Component Summary; 6M: 6 months; 12M: 12 months; SD: standard deviation.

Higher scores on MSIS-29 indicate worse HRQOL, and higher scores on the SF-36 indicate better HRQOL.

### Confirmed and unconfirmed significant PROM change over time

**Figure 3.**
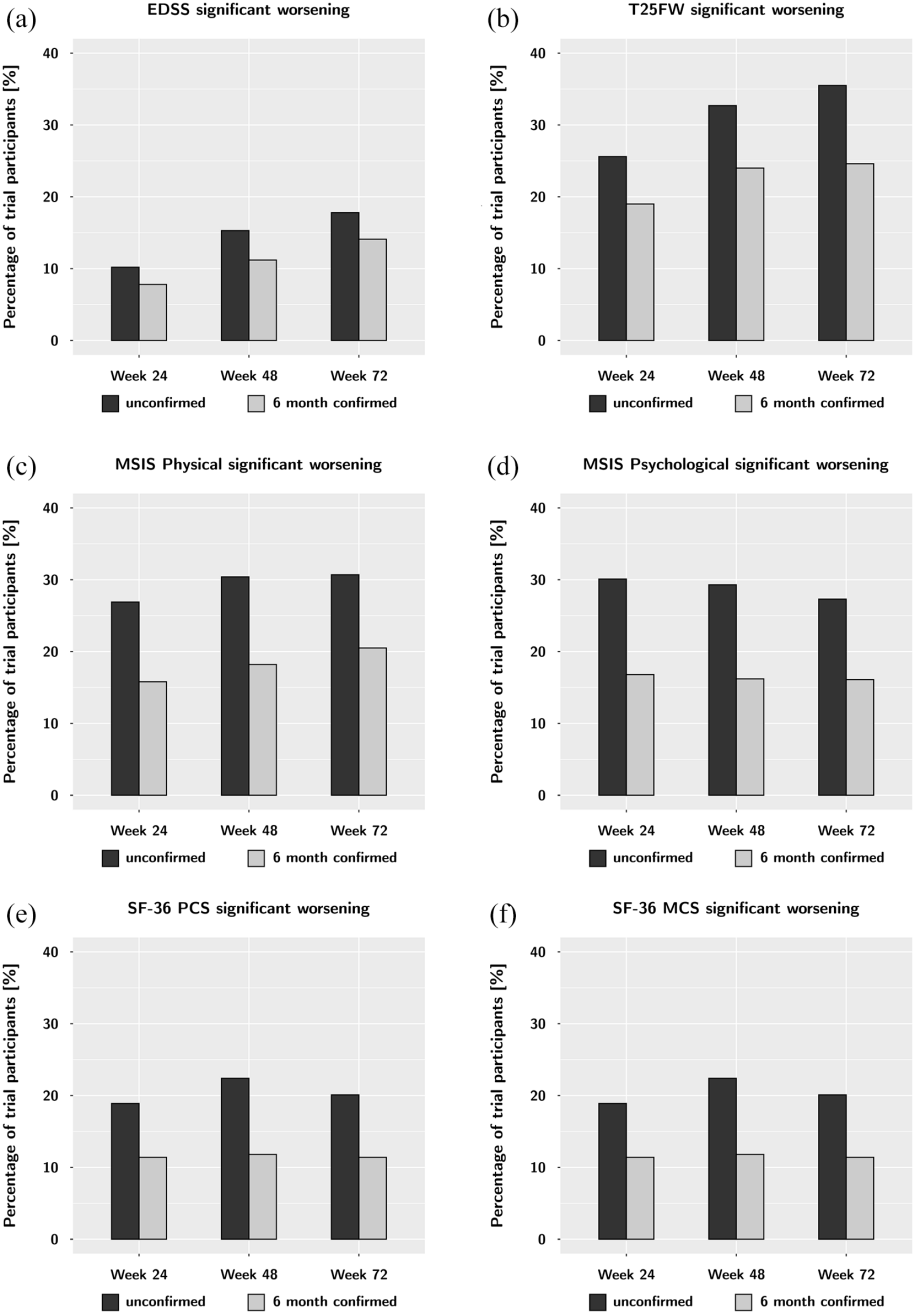
Unconfirmed and 6-month confirmed worsening on the EDSS (a), T25FW (b), and the four HRQOL summary scores: MSIS Physical (c), MSIS Psychological (d), SF-36 PCS (e), and SF-36 MCS (f) over the course of the trial. While 6-month confirmation always reduces the number of worsening events, this effect is much more pronounced in for the HRQOL summary scores. This argues for an increased variability of these measures over time.

Using the concept of ‘confirmed change’, we substantially reduced the percentages of participants with significant worsening and improvement. Using 6 months confirmation, the number of participants with significant worsening on the MSIS Physical decreased from 26.9% (unconfirmed) to 15.8% (6 months confirmed) at week 24 (Table 2 and Figure 3). A similar change was seen for proportions of improvement; the proportion of significant improvement on the MSIS Physical decreased from 31.8% (unconfirmed) to 20.2% (6 months confirmed) at week 24. These meaningful reductions were also seen at later time points and were similar to those for other PROM summary scores. Using 12-month confirmation did not change these proportions substantially (Table 2). The physical outcomes of EDSS and T25FW were less affected by 6-month confirmation (Table 2 and Figure 2).

### Additional analysis: PROM worsening in later time intervals

In our analyses of the proportion of participants with significant worsening from baseline, we found a remarkably large jump in worsening events from baseline to 24 weeks, but little change after week 24 (Table 2, Figure 2). To study whether there is a specific significance to this early period in the trial, we also determined the proportion of patients with significant HRQOL change between 24 and 48, 48 and 72, and 72 and 96 weeks (Table 3). The proportion of patients with significant worsening or improvement was strikingly similar in each epoch, suggesting that the large ‘jump’ in both worsening and improvement events between baseline and week 24 is due to the variability of the measures, rather than a specific event in the early phase of the trial.

**Table 3.** Proportion of patients with significant change (worsening or improvement) in HRQOL measures over 2 years of follow-up compared to the previous visit ( ‘rebaselined’).

Outcome				
Participants with unconfirmed (U) worsening or improvement (%) compared to previous visit	24 weeks vs baseline	48 vs 24 weeks	72 vs 48 weeks	96 vs 72 weeks
MSIS-29 Physical worse U (*n* (%))	210 (26.9)	204 (28.5)	190 (28.4)	157 (24.3)
MSIS-29 Physical better U (*n* (%))	248 (31.8)	176 (24.5)	143 (21.4)	125 (19.4)
MSIS-29 Psychological worse U (*n* (%))	239 (30.1)	221 (30.2)	192 (28.2)	183 (28.3)
MSIS-29 Psychological better U (*n* (%))	289 (36.4)	226 (30.8)	202 (29.7)	199 (30.8)
SF-36 PCS worse U (*n* (%))	139 (18.9)	133 (19.6)	115 (18.3)	106 (17.6)
SF-36 PCS better U (*n* (%))	158 (21.4)	113 (16.7)	99 (15.7)	89 (14.8)
SF-36 MCS worse U (*n* (%))	175 (23.7)	174 (25.7)	153 (24.3)	161 (26.8)
SF-36 MCS better U (*n* (%))	196 (26.6)	161 (23.8)	148 (23.5)	142 (23.6)

MSIS: Multiple Sclerosis Impact Scale; PCS: Physical Component Summary, MCS: Mental Component Summary.

Higher scores on -29 indicate worse HRQOL, higher scores on the -36 indicate better HRQOL.

To investigate the association of change in PROMs and disability worsening in these intervals, we compared the change in summary PROM scores between participants with and without significant EDSS and T25FW worsening. Participants with significant EDSS and T25FW worsening generally had scores suggestive of worse HRQOL, although these differences only rarely reached statistical significance (Table 4).

**Table 4.** Mean change in HRQOL measures by unconfirmed significant EDSS and T25FW worsening over 2 years of follow-up compared to the previous visit ( ‘rebaselined’). Participants with worsening physical disability generally had score changes suggestive of worse HRQOL. These differences occasionally reached statistical significance.

			24 weeks vs baseline		48 vs 24 weeks		72 vs 48 weeks		96 vs 72 weeks	
			Scores	p	Scores		Scores		Scores	
MSIS-29 Physical score change (mean, SD)	EDSS worsening	no	–1.46 (17.51)	0.07	0.12 (15.15)	0.21	0.19 (14.92)	0.27	0.61 (13.74)	0.07
		yes	1.90 (22.12)		6.46 (17.7)		5.05 (14.61)		4.07 (15.43)	
MSIS-29 Psychological score change (mean, SD)	EDSS worsening	no	–1.92 (19.04)	0.05	–1.01 (17.49)	0.8	–0.96 (16.82)	0.45	–0.25 (16.0)	0.8
		yes	3.48 (22.40)		6.96 (19.73)		3.95 (17.57)		4.31 (19.35)	
Mean SF-36 PCS score change (mean, SD)	EDSS worsening	no	0.32 (6.42)	0.50	**–0.10 (5.81)**	**0.04**	0.16 (5.95)	0.40	0.11 (5.30)	0.44
		yes	0.0 (7.27)		**–2.33 (6.49)**		–1.67 (5.50)		–1.21 (6.19)	
Mean SF-36 MCS score change (mean, SD)	EDSS worsening	no	0.48 (8.93)	0.58	–0.06 (8.51)	0.11	–0.17 (8.10)	0.32	0.06 (8.00)	0.17
		yes	–1.00 (9.20)		–3.48 (10.21)		–0.74 (7.37)		–3.00 (8.33)	
MSIS-29 Physical score change (mean, SD)	T25FW worsening	no	**–2.90 (17.13)**	**0.04**	–0.16 (14.70)	0.30	–0.45 (14.82)	0.34	0.63 (12.14)	0.09
		yes	**2.86 (19.31)**		4.23 (16.22)		3.68 (14.18)		4.31 (15.84)	
MSIS-29 Psychological score change (mean, SD)	T25FW worsening	no	**–2.67 (18.54)**	**0.03**	–0.83 (17.46)	0.68	–1.51 (16.38)	0.37	0.10 (14.83)	0.90
		yes	**1.73 (21.21)**		2.43 (18.88)		3.08 (17.55)		1.34 (19.28)	
Mean SF-36 PCS score change (mean, SD)	T25FW worsening	no	0.73 (6.39)	0.97	**0.05 (6.01)**	**0.01**	0.24 (5.81)	0.23	–0.09 (5.31)	0.25
		yes	–0.58 (6.68)		**–1.52 (5.65)**		–1.06 (6.09)		0.16 (5.79)	
Mean SF-36 MCS score change (mean, SD)	T25FW worsening	no	1.06 (8.69)	0.93	–0.23 (8.54)	0.06	–0.14 (8.01)	0.21	–0.06 (7.78)	0.11
		yes	–1.42 (9.23)		–0.88 (9.52)		–0.66 (8.12)		–1.25 (8.48)	

MSIS: Multiple Sclerosis Impact Scale; EDSS: Expanded Disability Status Scale; PCS: Physical Component Summary, MCS: Mental Component Summary.

Higher scores on MSIS-29 indicate worse HRQOL, higher scores on the SF-36 indicate better HRQOL.

### Threshold definitions of significant PROM change

To explore the influence of the threshold definition for significant change on worsening and improvement percentages, we repeated the analysis using different threshold definitions for each of the PROMs (any, 4-, and 16-point change for MSIS-29, and any, 2-, and 10-point change for SF-36). Using different cut-offs for defining significant change did not change the overall balance between worsening versus improvement events. In general, similar proportions of patients worsened or improved using any of the explored thresholds (Table 5).

**Table 5.** Worsening and improvement proportions with different thresholds for change on the PROMs.

	24 weeks	48 weeks	72 weeks	96 weeks
MSIS-29 Physical any worsening (*N*(%))	368 (47.2)	342 (47.6)	318 (47.6)	323 (51.0)
MSIS-29 Physical any improvement (*N*(%))	381 (48.8)	344 (47.9)	319 (47.8)	290 (45.8)
MSIS-29 Physical 4 points worsening (*N*(%))	283 (36.3)	272 (37.9)	260 (38.9)	269 (42.5)
MSIS-29 Physical 4 points improvement (*N*(%))	313 (40.1)	289 (40.3)	262 (39.2)	243 (38.4)
MSIS-29 Physical 16 points worsening (*N*(%))	108 (13.8)	120 (16.7)	116 (17.4)	140 (22.1)
MSIS-29 Physical 16 points improvement (*N*(%))	141 (18.1)	132 (18.4)	130 (19.5)	117 (18.5)
MSIS-29 Psychological any worsening (*N*(%))	333 (42.0)	315 (42.7)	276 (40.8)	257 (40.1)
MSIS-29 Psychological any improvement (*N*(%))	393 (49.6)	353 (47.9)	337 (49.8)	346 (54.0)
MSIS-29 Psychological 4 points worsening (*N*(%))	274 (34.6)	265 (36.0)	230 (34.0)	228 (35.6)
MSIS-29 Psychological 4 points improvement (*N*(%))	349 (44.0)	309 (41.9)	288 (42.5)	300 (46.8)
MSIS-29 Psychological 16 points worsening (*N*(%))	131 (16.5)	118 (16.0)	110 (16.2)	109 (17.0)
MSIS-29 Psychological 16 points improvement (*N*(%))	169 (21.3)	162 (22.0)	149 (22.0)	158 (24.6)
SF-36 PCS any worsening (*N*(%))	349 (47.4)	351 (51.6)	316 (50.3)	301 (50.6)
SF-36 PCS any improvement (*N*(%))	382 (51.8)	329 (48.4)	312 (49.7)	294 (49.4)
SF-36 PCS 2 points worsening (*N*(%))	239 (32.4)	253 (37.2)	241 (38.4)	227 (38.2)
SF-36 PCS 2 points improvement (*N*(%))	280 (38.0)	252 (37.1)	238 (37.9)	228 (38.3)
SF-36 PCS 10 points worsening (*N*(%))	43 (5.8)	46 (6.8)	36 (5.7)	33 (5.5)
SF-36 PCS 10 points improvement (*N*(%))	45 (6.1)	50 (7.4)	46 (7.3)	43 (7.2)
SF-36 MCS any worsening (*N*(%))	343 (46.5)	326 (47.9)	304 (48.4)	275 (46.2)
SF-36 MCS any improvement (*N*(%))	388 (52.6)	354 (52.1)	324 (51.6)	320 (53.8)
SF-36 MCS 2 points worsening (*N*(%))	263 (35.7)	259 (38.1)	247 (39.3)	212 (35.6)
SF-36 MCS 2 points improvement (*N*(%))	305 (41.4)	288 (42.4)	266 (42.2)	262 (44.0)
SF-36 MCS 10 points worsening (*N*(%))	92 (12.5)	87 (12.8)	77 (12.3)	70 (11.8)
SF-36 MCS 10 points improvement (*N*(%))	94 (12.8)	95 (14.0)	87 (13.9)	77 (12.9)

MSIS: Multiple Sclerosis Impact Scale; PCS: Physical Component Summary, MCS: Mental Component Summary.

### Association of baseline characteristics and significant PROM worsening

In our investigation of the association of PROM worsening with sex, EDSS at baseline and treatment arm, only female sex was significantly associated with worsening on the MSIS-psychological score (Table 6). We found no other significant associations.

**Table 6.** Contingency tables of significant PROM worsening by baseline disability status, sex, and treatment arm.

		EDSS			p*	Sex		p*	Trial arm		p*
		3.0 – 5.5	6.0	6.5		Female	Male		Placebo	NTZ	
MSIS-29 Physical	No worsening (*n*) – week 48	202	152	146	0.27	319	181	0.34	247	253	0.76
	worsening (*n*) – week 48	75	69	74		131	87		105	113	
	No worsening (*n*) – week 96	167	134	129	0.57	277	153	.65	210	220	0.9
	worsening (*n*) – week 96	72	62	69		127	76		98	105	
MSIS-29 Psychological	No worsening (*n*) – week 48	195	159	167	0.35	**341**	**180**	**0.004**	253	268	0.9
	worsening (*n*) – week 48	90	68	58		**117**	**99**		106	110	
	No worsening (*n*) – week 96	165	134	147	0.41	**301**	**145**	**0.001**	221	225	0.5
	worsening (*n*) – week 96	77	64	54		**106**	**89**		91	104	
SF-36 PCS	No worsening (*n*) – week 48	205	157	166	0.24	333	195	0.31	268	260	0.19
	worsening (*n*) – week 48	66	49	37		89	63		68	84	
	No worsening (*n*) – week 96	182	137	146	0.57	301	164	0.32	231	234	0.69
	worsening (*n*) – week 96	50	44	36		78	52		62	68	
SF-36 MCS	No worsening (*n*) – week 48	206	154	148	0.74	322	186	0.22	247	261	0.48
	worsening (*n*) – week 48	65	52	55		100	72		89	83	
	No worsening (*n*) – week 96	165	140	142	0.19	283	164	0.73	215	232	0.33
	worsening (*n*) – week 96	67	41	40		96	52		78	70	

EDSS: Expanded Disability Status Scale; NTZ: natalizumab; MSIS: Multiple Sclerosis Impact Scale; PCS: Physical Component Summary, MCS: Mental Component Summary.

* Chi-square test.

## Discussion

In this cohort of people with steadily worsening physical disability, PROM scores showed little consistent change. Our investigation showed that participants were roughly as likely to worsen on these measures as they were to improve over the course of 2 years of follow-up. The lack of change in these outcomes stands in contrast to the physical disability measures EDSS and T25FW, which show a steady increase in worsening events. These findings are somewhat unexpected since the MSIS-29 and SF-36 are well-validated scales of HRQOL in MS and reflect functional impairment in cross-sectional studies. The MSIS-29 has good test–retest reliability,^10^ and shows convergent validity with the EDSS.^11^ In a previous investigation of the ASCEND data set, we found an association of disability worsening, especially on the T25FW and EDSS, with MSIS-29 and SF-36.^14^ Based on these data, we had expected PROM summary scores to steadily worsen, and that worsening events would occur more frequently than improvement events. We chose the T25FW and EDSS as comparators because they are reliable measures of physical disability worsening in SPMS based on previous studies,^15,16^ but it should be kept in mind that both of these measures rely on ambulation. It is possible that the investigated PROMs are a better measure of other physical or mental functional domains that are not well quantified by the T25FW and EDSS.

One explanation for the similar proportions of worsening and improvement events may be that the MSIS-29 and SF36 are simply not responsive enough to detect the clinical progression present in the specific population in this cohort. Much of the research on the psychometric properties of the MSIS-29 and SF-36 in MS comes from cross-sectional studies.^2,17–26^ Such studies showed a significant association between the physical PROM subscores and disability. However, this does not prove the usefulness of these measures as longitudinal outcome measures in a clinical trial. There are only a few studies of the responsiveness of longitudinal measurements of the MSIS-29 and SF-36 in MS.^9,17,19^ Ideally, responsiveness is based on one gold standard measuring the same construct^26,27^ but often it is defined based on a change in a reference measure that represents clinical or performance status or global perceived effect after an intervention.^27,28^ For the MSIS-29 Physical subscore, the minimal clinically important difference (MCID) score of 8 was determined and validated using predefined significant worsening on the EDSS.^8,9^ To the best of our knowledge, no study has determined a specific MCID of the SF-36 in MS. Therefore, most often the value of one half of the standard deviation of a healthy standard population (which was 5 for the SF-36) is used.^10,11^ This approach is far from ideal since it is heavily dependent on the variance in the (healthy) reference population and may not reflect the changes MS patients find clinically relevant.

It is also unclear whether it is appropriate to use a single MCID definition for a heterogeneous disease such as MS. The sensitivity for detecting change can differ between disability strata or disease courses, and the population in which an MCID is used should always match the population in which the MCID was determined. For the MSIS-29, there is a different responsiveness in people with higher compared to lower disability: the MSIS-29 tends to perform better in higher disability strata (EDSS 5.5-8.5).^9^ However, in patients with a higher EDSS, the response shift phenomenon introduces yet another type of variation, as patients with high EDSS scores tend to score better on the MSIS-29 measures over time based on a different appreciation of the impact of disability.^29^ This effect results in improved scores in the absence of a change in functioning level. PROMs not only reflect physical limitations, but also psychological factors, resilience, and physical or psychological adaptations to changing physical abilities. Those who adapt to worsening physical limitations may retain similar scores on PROMs. Our investigation showed similar rates of worsening and improvement at a variety of thresholds, both lower and higher than those currently used. This suggests that a useful definition of significant change in the investigated PROMs may not exist.

Another aspect of the variation in PROMs can be seen in the comparison of unconfirmed and confirmed significant worsening. Most worsening events on an ideal outcome that measures ‘fixed’ change in HRQOL should persist over time, so that the difference between unconfirmed and confirmed worsening events should be small. In ASCEND, the number of events substantially decreased after introducing confirmation. A previous study in a community population of people with MS showed a large standard error of measurement with a relatively broad 95% confidence interval for individual MSIS-29 physical scores (SEM 5.0, 95% CI +/- 9.8).^17^ This implies that individual variation may exceed the MCID, introducing important problems for longitudinal studies that depend on the MCID as a threshold for significant change. Indeed, an investigation in a clinical cohort of people with MS showed that conventional PROMs for HRQOL in MS, including the SF-36 and MSIS-29, correlate well with the EDSS and T25WF cross-sectionally, but correlations between longitudinal changes in disability measures and PROMs were low, suggesting low reliability to detect disability worsening.^30^

Even though the main focus of this study was not to investigate factors contributing to HRQOL change, we did analyse some baseline factors that could have influenced the changes in PROM summary scores. Unfortunately, we were not able to include reliable measures of depression and fatigue, which often have an effect on HRQOL.^31^ Analysis of other potential influencing factors such as treatment-arm, sex, and disability status showed that sex was associated with worsening in the MSIS-29 psychological subdomain. While the treatment arm was not associated with significant differences in the investigated PROMs in this study, natalizumab treatment was reported to have a positive effect on SF-36 summary scores in the AFFIRM and SENTINEL trials in relapsing-remitting MS.^32^ It would be worthwhile to investigate longitudinal change in PROMs and the effect of treatment on HRQOL in relapsing-remitting MS cohorts.

A major strength of this study is its grounding in a clinical trial where systematic measurements and assessments were carried out. A potential limitation is the over 20% dropout rate that impacted both treatment arms. This may have dampened the changes in HRQOL if those who experienced the most changes systematically withdrew. While this could be an explanation for why people quit the study, the analyses of the incremental time points should have shown more robust changes if this were the case.

Taken together, the issues with responsiveness, and the lack of longitudinal correlations with physician and performance-based outcome measures across the different disability strata inherent in the investigated HRQOL-related PROMs, make changes on these measures difficult to interpret. Given our results, significant change in the SF-36 and MSIS-29 as currently defined should not be used to inform clinical decision making. The use of these PROMs as primary longitudinal outcome measures in SPMS clinical trials is untimely. Further research is necessary to determine which PROMs to use and how to define meaningful change on such measures in clinical trials in SPMS.
